# The Rice BZ1 Locus Is Required for Glycosylation of Arabinogalactan Proteins and Galactolipid and Plays a Role in both Mechanical Strength and Leaf Color

**DOI:** 10.1186/s12284-020-00400-9

**Published:** 2020-06-17

**Authors:** Sitong Liu, Yijun Tang, Nan Ruan, Zhengjun Dang, Yuwei Huang, Wei Miao, Zhengjin Xu, Fengcheng Li

**Affiliations:** Key Laboratory of Crop Physiology, Ecology, Genetics and Breeding, Ministry of Agriculture, Shenyang Agricultural University, Shenyang, China

**Keywords:** Mechanical strength, Leaf color, UDP-galactose/glucose epimerase, Cell wall, AGPs, MGDG, Chloroplast

## Abstract

**Background:**

The cell wall and chloroplast are two fundamental structures determining plant mechanical strength and grain yield. Therefore, understanding mechanisms that improve plants’ ability to develop a robust cell wall and well-developed chloroplast is of utmost importance for agricultural activities.

**Results:**

In this study, we report the functional characterization of a novel rice mutant, brittle stem and zebra leaf (*bz1*), which displays altered cell wall composition and collapsed chloroplast membrane. Molecular and biochemical analysis revealed that BZ1 encodes a functional UDP-galactose/glucose epimerase (UGE) and is ubiquitously expressed with higher expression in stem and leaf tissues. Multiple techniques analyses, including immunoblots, immuno-gold, and cryogenic scanning electron microscopy, demonstrated a significantly impaired glycosylation of arabinogalactan proteins (AGPs) and disordered cellulose microfibril deposition in *bz1*. Lipid profiling assay showed that the amount of monogalactosyldiacylglycerols (MGDG), a major chloroplast membrane glycolipid, was significantly decreased in *bz1*. Taken together, these results strongly demonstrate that BZ1 participates in UDP-galactose supply for the sugar chains biosynthesis of AGPs and MGDG, which thereby, respectively, results in altered cell wall and abnormal chloroplast development. Due to inferior mechanical strength and reduced photosynthesis, *bz1* plants displayed detrimental agronomic traits, whereas BZ1 overexpressing lines showed enhanced plant growth. Transcriptome analysis of stems and leaves further showed that numerous key genes involved in AGPs biosynthesis and photosynthesis metabolism were substantially suppressed in *bz1*.

**Conclusions:**

Our finding identifies BZ1 as a dual-targeting UGE protein for glycosylation of AGPs and MGDG and suggests a strategy for breeding robust elite crops.

## Background

The cell wall and chloroplast are two important structures that distinguish plants from animals and play essential roles in determining cereal plant architecture and grain yield (Somerville, 2006; Höfte and Voxeur, 2017; Kirchhoff, 2019). The plant cell wall not only protects cells against various environmental stressors but also presents the most abundant natural resource for biofuel production (Somerville et al., 2004; Cosgrove, 2005; Pauly and Keegstra, 2010; Bartley et al., 2013). The chloroplast is a primarily photosynthesis associated organelle determining yield and quality of crops (Tanaka and Tanaka, 2006). Although progress has been made in the characterization of genes involved in either cell wall biosynthesis or chloroplast development individually, no gene simultaneously contributing to both of these processes has been revealed so far.

The plant cell wall is a dynamic and complex load-bearing network, which is mainly composed of polysaccharides, aromatic substances, and glycoproteins (Somerville et al., 2004). Although much progress has been made in the characterization of genes involved in the biosynthesis and regulation of cellulose, hemicellulose, and lignin (Ralph et al., 2004; Burton et al., 2006; Scheller and Ulvskov, 2010; McFarlane et al., 2014; Kumar et al., 2016), the mechanism(s) that synthesize the wall glycoproteins and their roles in cell wall biosynthesis and assembly have not yet been elucidated. Arabinogalactan proteins (AGPs), the most abundant glycoproteins in the cell wall, are highly glycosylated (Showalter, 1993). More than 90% of their total molecular mass comes from glycan moieties consisting of 1, 3-β-galactan and 1, 6-β-linked galactan chains, which are thought to be important for the functional diversity of AGPs (Ellis et al., 2010; Showalter et al., 2010; Knoch et al., 2013). In rice, a total of 69 genes have been predicted to be associated with AGPs biosynthesis (Ma et al., 2014); however, none of them have been genetically verified by characterization of mutants or transgenic plants.

The chloroplast contains a large amount of membranes, in which glycolipids are the most abundant constituent after proteins (Chaal and Green, 2005). Monogalactosyldiacylglycerol (MGDG) and digalactosyldiacylglycerol (DGDG) are two of the most abundant glycolipids present in thylakoid membranes and photosynthesis system I and II, which are crucial for photosynthetic efficiency and plant development (Mullineaux, 1999). The amount of MGDG and DGDG comprise approximately 75% of the total membrane lipid (Barber and Gounaris, 1986; Ohlsson et al., 1998). Three MGDG biosynthesis-associated genes, *MGD1/2/3*, have been characterized in *Arabidopsis* (Murata et al., 1990). Lesion in *MGD1* causes a lower MGDG level and abnormal chloroplast development, resulting in a complete impairment of photosynthetic efficiency (Jarvis et al., 2000; Kobayashi et al., 2007; Aronsson et al., 2008). Although glycolipids in the chloroplast membrane are highly glycosylated, the mechanism controlling their glycosylation has not yet been elucidated.

UDP-galactose (UDP-Gal) is an essential nucleotide-activated sugar donor required for the biosynthesis of heteroxylans, glycoproteins, and glycolipids (Verbančič et al., 2018). Despite the important role of UDP-Gal, the mechanism underlying its biosynthesis, flux, and distribution remains unclear. UDP-galactose/glucose epimerases (UGEs) have been reported to be involved in the bioconversion of UDP-Gal and UDP-Glc (Barber et al., 2006; Zhang et al., 2006; Rösti et al., 2007; Beerens et al., 2015). Five UGE isoforms have been identified in *Arabidopsis*, AtUGE2 and AtUGE4 participate in vegetative growth and cell wall biosynthesis, whereas AtUGE3 is specialized for pollen development, and AtUGE1 and AtUGE5 are respond to environmental stress (Seifert et al., 2002; Seifert et al., 2004; Rösti et al., 2007). Despite significant contributions of each gene to total UGE activity and the importance of *AtUGE* in various aspects of plant growth, no obvious morphological phenotypes have been observed in any single *Atuge* mutant grown on soil (Rösti et al., 2007). The *Arabidopsis uge2,4* double mutant shows dramatic growth defects, displaying an obvious reduction in rosette size and a serious delay in development, while other mutant combinations were partially aberrant (Rösti et al., 2007). Immunochemical analysis using specific monoclonal antibody reveals defects in secondary hypocotyl thickening and alterations of AGPs carbohydrate structure in hypocotyls of *uge2,4* mutants (Rösti et al., 2007). The rice genome encodes four putative UGE proteins; however, none of them have been genetically characterized due to lack of mutants.

Here, we report a novel rice mutant, *bz1*, that displays both brittle culm and zebra leaf phenotypes. The *bz1* mutant harbors a lesion in UGE, which reduces galactose supply for the sugar chains biosynthesis of AGPs and MGDG. The substantially reduced AGPs and MGDG result in altered cell wall composition and defective chloroplast structure, respectively, which further impacts mechanical strength and leaf color. In the present study, we elucidated the mechanism through which BZ1 participates in cell wall formation and chloroplast development. Manipulating this mechanism may enhance both mechanical strength and photosynthetic efficiency of plants and thus have applications in crop breeding.

## Results

### The *bz1* Mutant Displays both Brittle Culm and Zebra Leaf Phenotypes

**Fig. 1:**
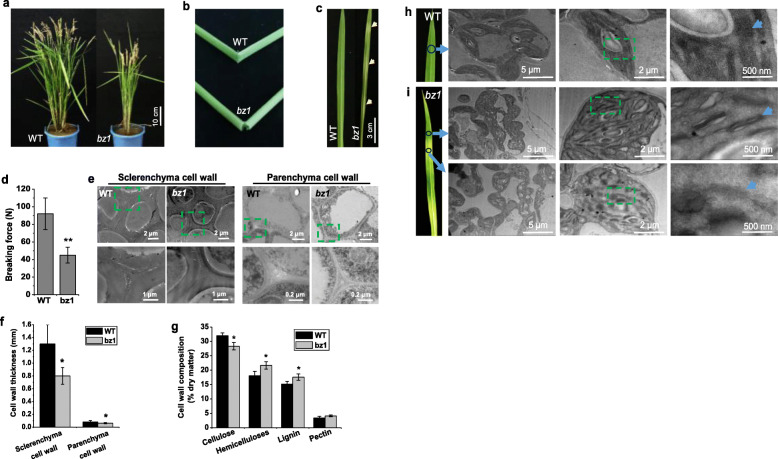
The *bz1* mutant displays both brittle culm and zebra leaf phenotypes. **a** Wild-type (WT) and *bz1* plants at the mature stage. The images are representative of 20 plants for each genotype. **b** Folding of stems of WT and *bz1* to show brittleness. **c** Leaf color of WT and *bz1* at the seeding stage. **d** Measurements of the breaking force (Newtons) of basal stem internodes. **e** TEM micrographs of sclerenchyma and parenchyma cells. **f** Measurement of cell wall thickness shows thinner walls in *bz1* compared with the WT. **g** Cell wall composition of mature stem. * indicates significant differences between WT and *bz1* by *t*-test at *P* < 0.05. **h-i** Chloroplast structures in leaves of WT (**h**) and *bz1* (**i**) at the tillering stage. The images are representative of six plants for each genotype

The mutant was named brittle culm and zebra leaf 1 (*bz1*) mainly based on its brittle culm and zebra leaf phenotypes under natural field growth conditions (Fig. 1a-c). The *bz1* mutant had a ~ 50% reduction in the stem breaking force compared with wild-type (Fig. 1d). As reduced mechanical strength usually results from altered cell wall properties (Aohara et al., 2009; Hirano et al., 2010; Zhang and Zhou, 2011; Li et al., 2017), we further analyzed the cell wall structure of wild-type and *bz1* culm internodes by transmission electron microscopy (TEM). The wall thickness of both sclerenchyma and parenchyma cells in *bz1* were obviously reduced, displaying an approximate 35% and 19% reduction, respectively, compared with that in wide-type plants (Fig. 1e and f). To explain the reduction in cell wall thickness of *bz1* plants, we examined the cell wall composition of comparable tissues harvested from the internodes of wild-type and *bz1* plants at the mature stage. Compared with the wild-type, the cellulose of *bz1* was significantly decreased, but the content of hemicelluloses and lignin were increased, and no significant differences were observed in the level of pectin (Fig. 1g).

The leaf color tends to be associated with chloroplast development. TEM analysis of one-month-old plant leaves revealed that the *bz1* displayed an abnormal chloroplast, including the altered chloroplast shape and significantly decreased number of thylakoid lamellar layers (Fig. 1h and i). Notably, the thylakoid membranes in *bz1* plants were obviously imperfect and even disrupted and disappeared, and the degree of membrane loss was proportional to the degree of leaf color (Fig. 1i).

### *BZ1* Encodes a Functional UGE and Is Broadly Expressed

**Fig. 2:**
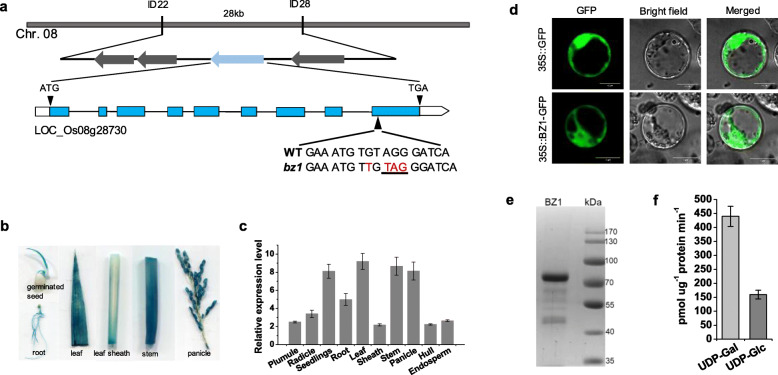
Molecular and biochemical identification of *BZ1*. **a** Map-based cloning of *BZ1*. The arrows indicate the candidate genes. The boxes and lines in the diagram of the *BZ1* gene indicate exons and introns, respectively. The arrowhead indicates a single base pair insertion (shown in a red-letter) in the last exon inducing a premature translational stop codon (underlined). **b** GUS activity staining assay in various organs of *BZ1pro::GUS* transgenic plants. **c** The expression pattern of *BZ1* detected by qRT-PCR. **d** Observation of BZ1-GFP in rice protoplast. Scale bar, 10 μm. **e** SDS/PAGE analysis of recombinant OsBZ1 expressed and purified from *E. coli*. Protein molecular mass standards (in KDa) are indicated on the right. The molecular mass of recombinant BZ1 is larger than the native BZ1 (due to the N-terminal GST tag fusion that facilitates purification). **f** UDP-Gal and UDP-Glc substrate specificity of recombinant purified BZ1. The error bar indicates SD values (*n* = 3)

To understand the molecular mechanism responsible for the *bz1* phenotypes, we used a map-based cloning approach to isolate the *BZ1* gene. By using 1260 F2 mutant plants generated from a cross between *bz1* and an *indica* cultivar Shennong265, the candidate gene was mapped to a 28-kb region on chromosome 8 via mapped-based cloning (Fig. 2a). Four putative open reading frames (ORFs) are annotated by TIGR Rice Genome Annotation Project (http://rice.plantbiology.msu.edu) in the 28-kb region. We sequenced and compared these with those of the wild-type. A single nucleotide insertion was found in the last exon of LOC_Os08g28730, which induces a premature translational stop codon (Fig. 2a). To validate the cloning result, a complementary vector containing LOC_Os08g28730 genomic sequence comprising the ORF and 1.8 kb upstream and 1.6 kb downstream regions derived from the wild-type was transferred into *bz1* plants. The transgenic plants rescued the mutant phenotypes, including both stem mechanical strength and leaf color (Figure S1b-d). Furthermore, we knocked out the LOC_Os08g28730 gene in the wild-type using CRISPR/Cas9 genome editing system and selected out the homozygous transgenic line *bz/cs1* that outcrossed the CRISPR/Cas9 construct. The *bz/cs1* harbored two nucleotide deletions in the third exon, which induced a premature translational stop codon (Figure S1a). The *bz/cs1* exhibited similar brittleness and zebra leaf phenotypes as *bz1* mutant (Figure S1c and d). These results confirm that the *bz1* gene is responsible for the brittle culm and zebra leaf phenotypes.

To examine the spatial and temporal expression patterns of *BZ1*, we generated transgenic plants expressing the β-*glucuronidase* (*GUS*) gene driven by *BZ1* promoter (*BZ1*_*pro*_*: GUS*) and detected the promoter activity of *BZ1* in various organs at distinct development stages (Fig. 2b). GUS signals were observed in all organs examined, with higher expression in the stem and leaf. We further confirmed these results using qRT-PCR (Fig. 2c). Therefore, *BZ1* is ubiquitously expressed, especially in the tissues that confer mechanical strength and chloroplast development in rice. To investigate the subcellular location of BZ1, we fused BZ1 with a green fluorescent protein (GFP) at the carboxyl terminus and transfected rice protoplasts with it. The resulting GFP signals indicated that BZ1 was localized to the cytosol (Fig. 2d).

Sequence alignment suggested that BZ1 is a member of UGE family protein (Figure S2), which participates in the bioconversion of UDP-Gal and UDP-Glc (Seifert et al., 2002; Barber et al., 2006; Rosti et al., 2007). The conserved UGE motif and catalytic residue indicated that BZ1 may have UDP-Gal/Glc epimerase activity. To test this, we expressed GST-tagged BZ1 recombinant protein in *E.coli* (Fig. 2e). The purified BZ1 protein was incubated with UDP-Glc and UDP-Gal, respectively, to determine if the protein could effectively catalyze the bioconversion of UDP-Gal and UDP-Glc. The result showed that BZ1 could catalyze the interconversion of UDP-Gal and UDP-Glc, displaying a higher enzymatic activity in the direction of UDP-Gal to UDP-Glc than that in the opposite direction (Fig. 2f).

### BZ1 Mutation Impairs AGPs Biosynthesis

**Fig. 3:**
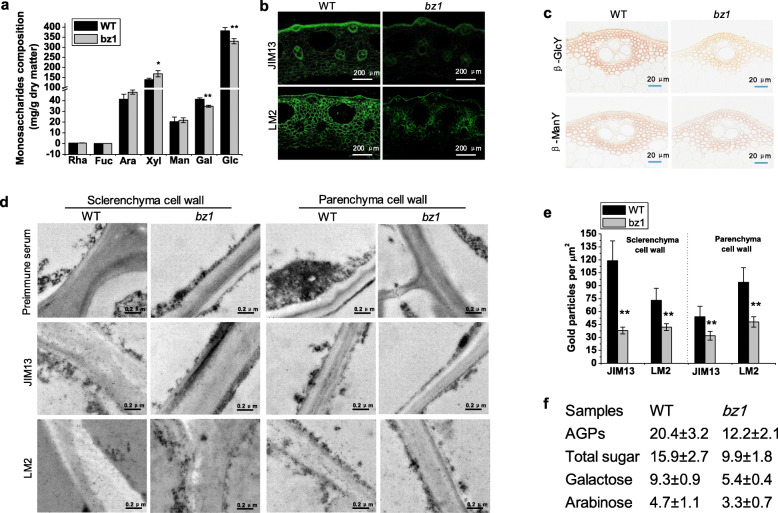
The *bz1* mutant displays impaired AGPs biosynthesis. **a** Comparison of monosaccharides composition of mature stem between wild-type (WT) and *bz1* plants. * and ** indicates significant differences between WT and *bz1* by *t*-test at *P* < 0.05 and 0.01, respectively. **b** Immunohistochemical analysis of AGPs in culm sections using JIM13 and LM2 monoclonal antibodies. The *bz1* shows substantially weaker signals than WT in all cells including the vascular bundle, sclerenchyma and parenchyma cells. **c** Staining of hand-cut stem sections with βGlcY and βManY (control). The *bz1* shows weaker signal than the WT. **d** Immunogold labeling of sclerenchyma and parenchyma cell walls of WT and *bz1* plants with JIM13 and LM2 monoclonal antibodies. **e** The *bz1* shows fewer particles in both sclerenchyma and parenchyma cell walls. **f** Comparison of the abundance of AGPs and its monosaccharides composition in WT and *bz1*. The AGPs content was measured and calculated as μg g^− 1^ fresh weight of tissue. The results are means ± SE of three independent assays

Since BZ1 catalyzed the bioconversion between UDP-Gal and UDP-Glc, we compared individual monosaccharides composition of cell wall between wild-type and *bz1* plants by GCMS. The *bz1* mutant showed significantly reduced glucose and galactose (*P* < 0.01) and an increased xylose level, in agreement with its lower cellulose content and higher hemicelluloses content (Fig. 3a). Given the AGPs are the most abundant cell wall associated glycoproteins containing large amounts of galactan chains, we, therefore, analyzed AGPs content using two monoclonal antibodies (JIM13 and LM2), which enabled us to compare the carbohydrate AG epitopes of AGPs between wild-type and *bz1* plants. Clear reductions in the labeling by JIM13 and LM2 were observed in all cells of *bz1* including vascular bundles, sclerenchyma, and parenchyma cells (Fig. 3b). Additionally, staining with β-glucosyl Yariv reagent (βGlcY), a chemical that is widely used to localize and quantify AGPs, also revealed a reduction in AGPs in hand-cut stem sections of *bz1* plants, but no difference was observed using β-mannosyl Yariv reagent (βManY), an inactive analogue of βGlcY (Fig. 3c).

To investigate the distribution of AGPs in cell walls, immuno-gold analysis was performed, and revealed that less labeling by JIM13 and LM2 was observed in both sclerenchyma and parenchyma cell walls of *bz1* plants (Fig. 3d and e). These results indicate that the deficiency in BZ1 affects AGP abundance in cell walls. The reduction of AGPs and galactose content in *bz1* leads us to examine the monosaccharides composition of AGPs. The content of AGPs in *bz1* was remarkably decreased, particularly, the galactose content in AGPs of *bz1* was decreased by 42% compared with that in the wild-type (Fig. 3f). These results strongly demonstrate that the BZ1 mutation perturbs the galactose supply to impair AGPs galactan chains and thereby to reduce AGPs content significantly.

### Lesion in BZ1 Results in Disordered Cellulose Deposition

**Fig. 4:**
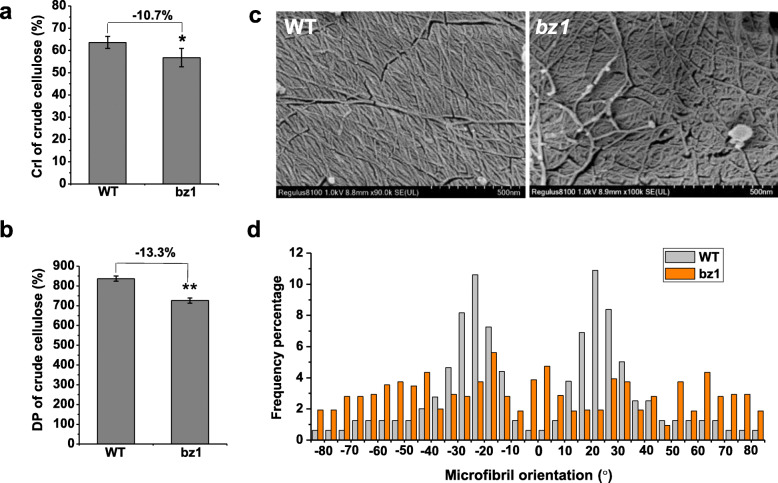
Detection of cellulose structural features. **a** Lignocellulose CrI of mature stems using the X-ray diffraction (XRD) method. **b** Cellulose DP of mature stems using the viscometry method. **c** Cryogenic scanning electron microscopy (Cryo-SEM) views of cellulose macrofibrils/microfibrils of the stem at booting stage after treatment with Updegraff reagent. **d** Distribution of macrofibrils/microfibrils orientation. The orientation of macrofibril/microfibril is represented as percentage frequency of the orientation of macrofibril/microfibril segments identified using the software Gwyddion (*n* = 3000 snakes from three images of three cells of three individual plants)

As AGPs have been reported to be involved in cell wall polymers assembly (Driouich and Baskin, 2008; MacMillan et al., 2010; Huang et al., 2013), and cellulose content is obviously reduced in *bz1* plants, we further investigated whether the cellulose features are changes in *bz1*. The crystallinity index (CrI) and degree of polymerization (DP), two important cellulose features, were significantly decreased in the mutant (Fig. 4a and b), indicating that BZ1 mutation affects the assembly and orientation of cellulose microfibrils. To confirm the altered cellulose features caused by BZ1 dysfunction, we further used cryogenic scanning electron microscopy (Cryo-SEM) to observe the cellulose microfibrils orientation in xylem cells of the wild-type and mutant mature internodes (Fig. 4c). In contrast to the wild-type cellulose microfibrils that were orderly orientated with a frequency of angle distribution of ±10 - 35°, the *bz1* plants displayed randomly orientated microfibrils (Fig. 4c and d). Taken together, the results show that the inferior mechanical strength of *bz1* probably results from aberrantly deposited cellulose microfibrils.

### The BZ1 Mutation Influences Biosynthesis of Galactolipids

**Fig. 5:**
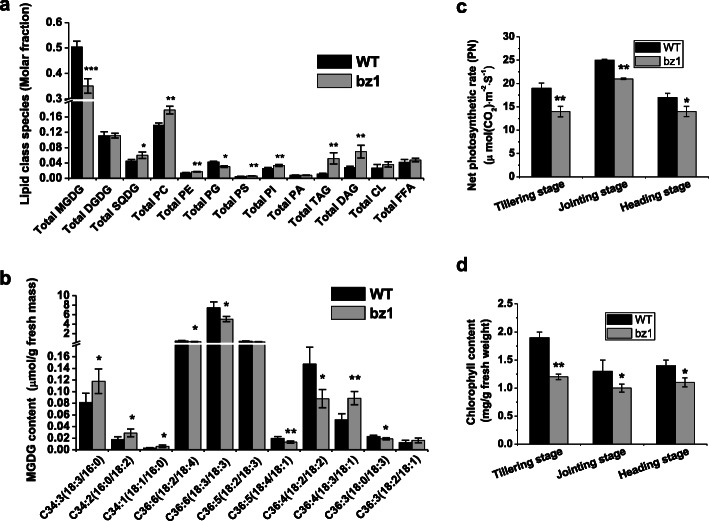
The BZ1 mutation influences biosynthesis of galactolipids and impairs photosynthesis efficiency and chlorophyll content. **a** Total lipid composition in leaves of wild-type (WT) and *bz1* plants grown in paddy fields. SQDG, sulphoquinovosyl diglyceride; PC, phosphatidylcholine; PE, phosphatidylethanolamine; PG, phosphatidylglycerol; PS, phosphatidylserines; PI, phosphatidylinositol; PA, phosphatidic acids; TAG, triacylglycerols; DAG, diacylglycerols; CL, cardiolipins; FFA, free fatty acids. **b** Fatty acid composition of MGDG from WT and *bz1* plant leaves. The results are means ± SE of five independent assays. **c-d** Detection of net photosynthetic rate (*P*_*N*_) **(c)** and chlorophyll content (**d**) of WT and *bz1* at three developmental stages, more than 20 representative leaves were selected for measuring. *, ** and *** indicates significant differences between WT and *bz1* by *t*-test at *P* < 0.05, 0.01 and 0.001, respectively

The chloroplast membrane has high levels of glycolipids, and UDP-Gal is a central nucleotide sugar donor for glycolipid biosynthesis. To explore the effect of BZ1 on chloroplast membrane lipid homeostasis, we analyzed the composition of total lipid extracted from leaves of wild-type and *bz1* by reverse phase high-performance liquid chromatography/electrospray ionization tandem mass spectrometry (RP-HPLC/ESI/MS/MS) (Fig. 5a). Significantly, the *bz1* mutant had an approximate 30% reduction in the MGDG level compared with wild-type, whereas no difference was detected for the amount of DGDG, another high level of glycolipid present in chloroplast membranes, in wild-type and *bz1*, demonstrating that BZ1 is probably involved in MGDG biosynthesis rather than DGDG. In addition, the reduced abundance of MGDG in *bz1* was accompanied by an increase in the abundance of other major membrane lipids such as triacylglycerols (TAG), diacylglycerols (DAG), sulfoquinovosyldiacylglycerol (SQDG), phosphatidylcholine (PC), phosphatidylinositol (PI), and phosphatidylethanolamine (PE), while the levels of cardiolipins (CL) and free fatty acids (FFA) were not altered significantly in the *bz1* mutant.

We further focused on the exclusive chloroplast lipid MGDG and compared the lipid molecular species of *bz1* with wild-type plants (Fig. 5b). After removing low-abundant species that are biologically ambiguous as to whether they are endogenously synthesized in rice, we detected a total of 11 fatty acid species. The C36:6(18:3/18:3) was the most abundant fatty acid species, which comprises approximately 85% of MGDG (Fig. 5b). The amount of C36:6(18:3/18:3) in *bz1* was reduced by 31% compared to that in wild-type, which might be the predominant contributor to its significantly reduced MGDG content. Additionally, C34:3(18:3/16:0), C34:2(16:0/18:2), and C36:4(18:3/18:1) containing polyunsaturated FAs were increased in *bz1* plants, but C36:6(18:2/18:4), C36:4(18:2/18:2), C36:5(18:4/18:1), and C36:3(18:0/18:3) were also markedly decreased (Fig. 5b). Taken together, these results strongly demonstrate that BZ1 catalyzes UDP-Gal biosynthesis to participate in the glycosylation of MGDG.

To investigate the effect of the zebra leaf phenotype on photosynthesis, we compared the photosynthesis of wild-type and *bz1* plants. The photosynthesis efficiency was significantly reduced in the leaves of *bz1* at all three developmental stages examined (Fig. 5c). Furthermore, the *bz1* plants showed an approximate 37% reduction in total chlorophyll content compared with that in wild-type plants at the tillering stage (Fig. 5d), coinciding with its lower photosynthesis efficiency.

### *BZ1* Influences Pleiotropic Agronomic Traits

**Fig. 6:**
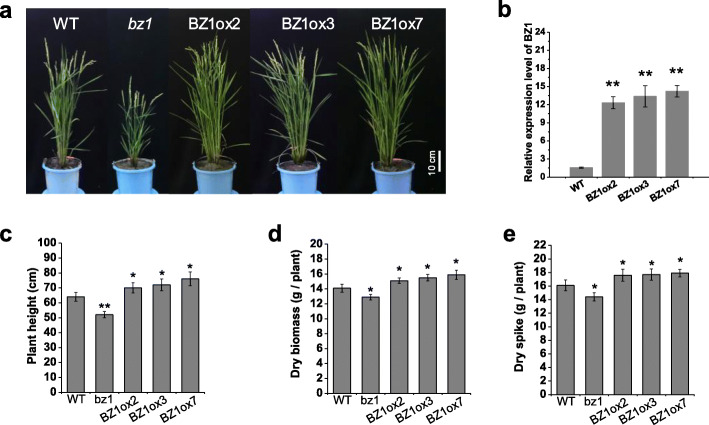
Agricultural traits of *bz1* plants and transgenic rice lines overexpressing BZ1. **a** Phenotypic differences of wild-type (WT), *bz1*, and transgenic line BZ1ox2, BZ1ox3, and BZ1ox7 at the grain-filling stage grown in paddy fields. **b** qRT-PCR analysis of the expression of *BZ1* in WT and *BZ1* overexpressing lines. **c-e** Plant height (**c**), dry biomass (**d**) and dry spike (**e**) were increased in transgenic plants overexpressing *BZ1*. * and ** indicates significant differences between WT and *bz1*, *BZ1* overexpressing lines by *t*-test at *P* < 0.05 and 0.01, respectively

Since lesion in BZ1 affects both cell wall properties and photosynthesis, we further evaluated the effects of BZ1 on plant growth by comparing the wild-type with *bz1* mutants and transgenic plants overexpressing BZ1 (Fig. 6a). Three independent lines that overexpressed *BZ1* were identified for further agronomic trait investigation (Fig. 6b). Compared to the wild-type, *bz1* mutants showed retarded growth, including reduced plant height, small panicle size and reduced tiller number, consequently reflected in significantly decreased biomass production and grain weight (Fig. 6c-e). In contrast, compared to non-transgenic control plants, transgenic rice plants overexpressing BZ1 showed a significant increase in grain yield and biomass production, by up to 11% and 12%, respectively, and the changes were consistent with the BZ1 expression levels (Fig. 6d and e). We further compared cell wall composition between wild-type and BZ1ox7, which shows the highest expression level of *BZ1* among those transgenic lines. Cellulose content was increased in the BZ1ox7 line, whereas the level of hemicellulose and lignin was not significantly altered compared with wild-type (Figure S3a). Measurement of chlorophyll content and photosynthetic rate of the top leaf at the tillering stage revealed that the BZ1ox7 has higher chlorophyll content and photosynthetic efficiency than wild-type (Figure S3b and c). These findings suggested that BZ1 overexpression in rice leads to enhancement in biomass production and grain yield by positively affecting cell wall biosynthesis and photosynthetic efficiency.

### Transcriptomic Changes in the Stem and Leaf of the *bz1* Mutant

**Fig. 7:**
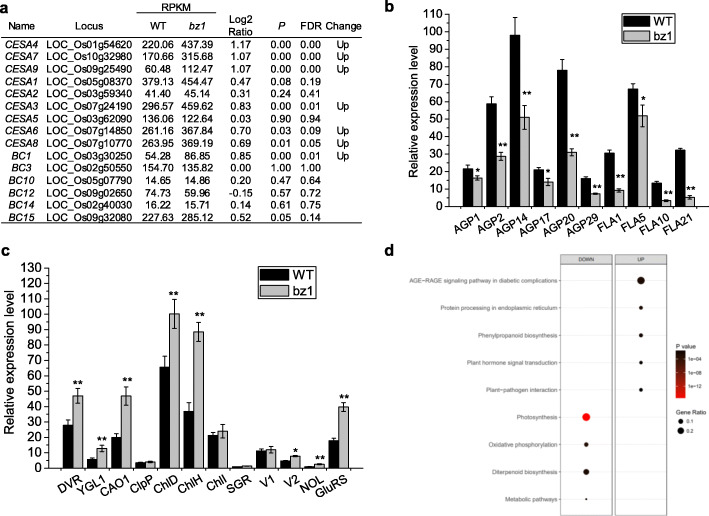
Transcriptome profile analysis of both stem and leaf tissues in wild-type (WT) and *bz1* by RNA-sequencing approach. **a** Expression levels of *CESA* (*Cellulose Synthase*) and *BC* (*Brittle Culm*) genes in WT and *bz1* plants based on stem RNA-sequencing analysis. (**b**) qRT-PCR analysis of AGPs biosynthesis related genes in second stem internodes of WT and *bz1* plants at the heading stage. **c** qRT-PCR analysis of representative genes involved in chloroplast development and chlorophyll biosynthesis using the top leaves of WT and *bz1* plants at the tillering stage. **d** KEGG enrichment analysis was performed to identify the altered pathways of differentially expressed genes (DEGs) based on leaves RNA-sequencing analysis. * and ** indicates significant differences between WT and *bz1* by *t*-test at *P* < 0.05 and 0.01, respectively

To gain insight into the effect of BZ1 on stem mechanical strength and leaf color, we performed RNA-sequencing (RNA-seq)-based comparative stems and leaves transcriptome analysis of wild-type and *bz1* plants (Fig. 7). A total of 4418 and 1353 differentially expressed genes (DEGs) were identified in stems and leaves, respectively. There were 1845 up-regulated and 2573 down-regulated transcripts in *bz1* stems compared with the wild-type, and 844 up-regulated and 509 down-regulated transcripts in *bz1* leaves (Figure S4a). As *BZ1* is a member of UGE family, we examined the expression level of four *UGE* genes in leaf tissues based on the RNA-seq analysis and qRT-PCR assay (Figure S4b and c). Both techniques were consistent to reveal that the *bz1* mutant had a significantly reduced expression level of BZ1 and increased expression of *PHD1*, which encodes a chloroplast-localized protein that possesses UGE activity and affects MGDG biosynthesis and photosynthetic efficiency in rice (Li et al., 2011).

Based on the considerable effects of the BZ1 mutation on cellulose content and stem mechanical strength, we examined the transcriptional levels of the *CESA* (*Cellulose Synthase*) and *BC* (*Brittle Culm*) genes contributing to both phenotypes using the stem transcriptomes analysis. The expression of both genes was unchanged or slightly increased in *bz1* (Fig. 7a). Notably, many genes putatively involved in AGPs biosynthesis were significantly down-regulated in *bz1* (Table S1). We verified the downregulation of AGPs biosynthesis-related genes in *bz1* by qRT-PCR (Fig. 7b).

Given that a lesion in *bz1* causes zebra leaf, a total of 88 previously characterized leaf color-related genes were examined using leaf transcriptomes analysis (Table S2). Despite the defective chloroplast in *bz1*, the expression level of most genes participating in chloroplast development, chlorophyll biosynthesis, and degradation were unchanged or even up-regulated in *bz1* (Table S2), indicating that *BZ1* mutation might trigger a feedback regulation to maintain the relatively normal function of the chloroplast in *bz1*. We further verified several representative leaf color-related genes by qRT-PCR. (Fig. 7c). Significantly, Kyoto Encyclopedia of Genes and Genomes (KEGG) enrichment analysis of leaf DEGs identified ‘Photosynthesis ko00195’ as the top annotated pathway (corrected *P*-value = 6.49E-15), in which a total of 21 downregulated genes were annotated as photosystem I- and II-associated proteins and ATP synthases (Fig. 7d and Table S3). The proteins encoded by these down-regulated genes putatively localize in the photosynthetic membrane system, coinciding with the defective chloroplast membrane in *bz1*.

## Discussion

In this study, we report on a novel rice mutant displaying both the brittle culm and zebra leaf phenotypes, which is distinct from previously identified brittle culm or leaf color-related mutants. Although alteration of AGPs epitopes have been observed in *Arabidopsis uge2,4* double mutants (Rösti et al., 2007), the rice BZ1 catalyzes galactose production for both cell wall formation and chloroplast development, which is different from the roles of UGEs in *Arabidopsis*. Thus, this work identified BZ1 as a novel dual-targeting UGE protein for simultaneously affecting both cell wall formation and chloroplast development.

### Hypothetical Model for the Effects of BZ1 on both Mechanical Strength and Zebra Leaf

**Fig. 8:**
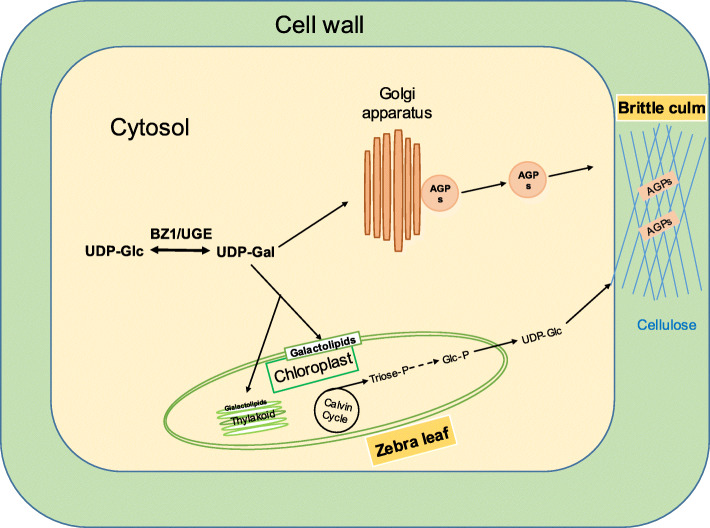
A hypothetical model for the role of BZ1 in the AGPs and galactolipid biosynthetic pathway for cell wall biosynthesis and chloroplast membranes formation, respectively

We propose a model to illustrate how a single BZ1/UGE protein controls both mechanical strength and leaf color in rice (Fig. 8). We present evidence that BZ1 has UGE activity and catalyzes the bioconversion of glucose and galactose. The BZ1 mutation impairs the biosynthesis of galactose, which is a principal monosaccharide for the polymerization of sugar chains of AGPs and MGDG. The defective AGPs caused by reduced galactose supply in *bz1* result in abnormal cellulose biosynthesis and deposition, which further leads to an altered cell wall composition and thereby significantly reduces mechanical strength. On the other hand, due to the lack of galactose supply, the MGDG is remarkably reduced in the leaf tissue of *bz1*, which results in a defective chloroplast membrane system and thus a zebra leaf phenotype. Furthermore, the defective chloroplast in *bz1* results in compromised photosynthesis and carbon fixation to impair cellulose biosynthesis by reducing UDP-Glc production, which should be another important contributor to the decreased mechanical strength.

### BZ1 Mutation Impairs AGPs

Galactose is a dominant glycosyl substrate for the biosynthesis of polysaccharides and glycoproteins carbohydrate chains. Since arabinoxylan is the most abundant hemicellulose in grasses and its content is higher in the *bz1* mutant, it is not likely that the galactose supplied by BZ1 is involved in hemicellulose formation. It is evident from our results that *BZ1* mutation impairs AGPs synthesis. Here, we have four pieces of evidence: 1 The *bz1* mutant has the reduction in cell wall thickness of both sclerenchyma and parenchyma, which coincides with the distribution of AGPs in the cell walls; 2 Multiple techniques, including immunolabelling, immuno-gold analysis and chemical staining, showed that AGPs abundance was reduced in *bz1*; 3 Determination of carbohydrate chains of AGPs by GCMS suggested that the reduction of AGPs in *bz1* was due to its deficient biosynthesis of galactan chain; 4 The stem RNA-seq assay revealed that numerous predicted AGPs biosynthesis-associated genes were down regulated in *bz1*.

### The Reduction of AGPs in *bz1* Causes its Abnormal Cellulose Deposition and Decreased Mechanical Strength

Plant mechanical strength is principally determined by cell wall composition. Although AGPs account for < 10% of the wall matrix, they have been reported to lead to lower mechanical strength by affecting the integrity of the cell wall (Schultz et al., 2004; Showalter et al., 2010). In *Arabidopsis*, insertional mutations in fasciclin-like AGPs (FLA) cause altered cell wall composition and reduced stem biomechanics (MacMillan et al., 2010). Defects in AGPs biosynthesis of *Eucalyptus* affect stem mechanics by altering secondary cell wall growth and properties (MacMillan et al., 2015). Consistently, the *bz1* plants displayed reduced mechanical strength and altered cell wall structure and composition with a significantly decreased AGPs level. Consistent with the report that AGP plays an important role in fibre initiation and elongation in cotton (Huang et al., 2013), the cellulose DP is significantly reduced in *bz1*. Furthermore, the *bz1* also shows remarkably disordered cellulose orientation and decreased cellulose CrI. This is consistent with the idea that AGPs might interact with the scaffold between microtubules and cellulose microfibrils, potentially affecting their orientation (Driouich and Baskin, 2008). Although the details about how AGPs structurally affect cellulose deposition remain unclear, our results suggest that impaired AGPs in *bz1* is the principal cause of its disordered cellulose deposition and that BZ1 is essential for the normal cellulose biosynthesis and maintenance of cell wall architecture.

### BZ1 Affects the Chloroplast Membrane System

Glycolipids are the second most abundant components after proteins in the chloroplast membrane system. Although glycosylation of lipids is essential for their functioning in an intact chloroplast membrane, the mechanism underlying it remains largely unknown. We provide evidences that BZ1/UGE2 mutation influences the galactan chain biosynthesis of MGDG to interrupt the normal chloroplast membrane, resulting in a zebra leaf. This is consistent with previous reports that *Arabidopsis* mutant *mgd1* lacking MGDG has disrupted photosynthetic membranes, leading to an impairment of photosynthetic ability and photoautotrophic growth (Jarvis et al., 2000; Kobayashi et al., 2007). A chloroplast-localized protein PHD1 was identified previously to have UGE activity and its mutation reduces chlorophyll content and photosynthetic activity by affecting the MGDG biosynthesis in rice (Li et al., 2011). In this study, however, the expression level of *PHD1* is up-regulated in the *bz1* mutant, which might trigger a feedback complementarity regulation to sustain the relative normal chloroplast membranes in *bz1*. As galactolipids synthase is estimated to be localized to the inner envelope membrane (Miège et al., 1999; Awai et al., 2001) and the UDP-Gal concentration is low within plastids (Bligny et al., 1990; Li et al., 2011), it is very likely that a large proportion of UDP-Gal required for synthesis of glycolipids in the chloroplast is mobilized from the cytosol. The BZ1 here is localized to the cytosol and displays UGE activity, indicating its important role in the UDP-Gal supply. MGDG is substantially reduced in *bz1*, whereas DGDG and non-glycosylated lipids did not show significant changes, strongly suggesting that BZ1 is crucial for the glycosylation of MGDG and thus influences the photosynthetic membrane system.

### BZ1 Affects the Expression of Genes Involved in AGPs Biosynthesis and Photosynthesis Pathway

The BZ1 mutation significantly downregulates the expression of genes involved in AGPs biosynthesis and photosynthesis, coinciding with the reduced AGPs content and impaired photosynthesis efficiency observed in *bz1* plants. Despite the reduced stem mechanical strength in *bz1*, the expression level of BC genes, which have been demonstrated to result in brittle culm phenotype in rice (Zhang and Zhou, 2011), are unchanged or even up-regulated in *bz1*, indicating that BZ1 influences mechanical strength through a different mechanism. As the cellulose features (CrI, DP) are altered and the AGPs content is significantly decreased in *bz1*, the BZ1 mutation probably regulates the cellulose deposition and mechanical property by downregulating AGPs biosynthesis-related genes expression and impairing AGPs distribution in the cell wall. Additionally, KEGG analysis of the DEGs showed a significant enrichment in the ‘photosynthesis’ pathway. Combined with the reduced cellulose content and imperfect photosynthetic membrane in *bz1*, these results strongly suggested that the BZ1 mutation might reduce the carbon source supply to impair cellulose biosynthesis by downregulating the expression of photosynthesis and carbon fixation and metabolism related genes.

### The BZ1 Mutation Causes Pleiotropic Phenotypes

The BZ1 mutation causes defective biosynthesis of AGPs and MGDG, which further impairs cell wall assembly and chloroplast development, respectively. Defects in cell wall structure and the chloroplast thereby result in abnormal plant growth. The *bz1* plants here displayed pleiotropic phenotypes, including brittle culm, reduced plant height and biomass production. In contrast, BZ1 overexpression might increase the amount of AGPs and MGDG, which facilitates cellulose deposition and thylakoid membranes synthesis, respectively, increasing biomass production and grain yield. These results strongly demonstrate the importance of BZ1 in plant growth and development, indicating its significant economic implications in both crop genetic improvement and bioenergy crop breeding.

## Conclusions

Mutation of rice BZ1, a novel functional UGE, impairs sugar chains biosynthesis of arabinogalactan proteins (AGPs) and monogalactosyldiacylglycerol (MGDG) by reducing UDP-galactose supply, which further impacts mechanical strength and leaf color, respectively. The improved agronomic traits in *BZ1* overexpressing lines suggest that manipulation of this gene can facilitate to breed robust elite rice varieties.

## Methods

### Plant Material and Growth Conditions

The *bz1* mutant was isolated from the EMS-induced mutation population of the early maturing cultivar kitaake. An F2 mapping population was generated from a cross between *bz1* and Shennong265, an *indica* cultivar. Rice plants were cultivated in the experimental station of the Shenyang Agricultural University in Shenyang in natural growing seasons.

### Measurements of Plant Mechanical Properties and Agronomic Traits

The breaking force of rice culm was determined using a digital force/length tester (RH-K300, Guangzhou, China). Rice dry spike and dry biomass were respectively weighed after the samples were dried in the oven at 60 °C. All measurements were conducted using nine independent biological duplicates.

### Map-Based Cloning

*BZ1* was mapped and cloned using 1260 F2 mutant plants with SSR and InDel molecular markers. The *bz1* was localized between the two new insertion/deletion marker ID22 (forward primer: gaccaaagccttgcacaatg; and reverse primer: actcaaaagacaaatgtagg) and ID28 (forward primer: agttggtgtgccaacgtgca; and reverse primer: cagtaatcctggccaacaac) on chromosome 8 within a 28 kb in the rice genome (Fig. 2a), which contains four ORFs annotated by TIGR Rice Genome Annotation Project (http://rice.plantbiology.msu.edu). The four genes were amplified from the mutants and their corresponding wild-type plants by PCR with KOD-Plus (TOYOBO, Japan) and sequenced with a 3730 sequencer (ABI, Massachusetts, USA). An insertion mutation was found in the last exon of LOC_Os08g28730.

### Generation of Transgenic Rice Plants

For complementation analysis, a 9.6 kb genomic DNA fragment containing the entire *BZ1* coding region, a 1.8 kb upstream sequence and a 1.6 kb downstream sequence were inserted into the binary vector pCAMBIA 1300 to generate the transformation plasmid. This binary plasmid was introduced into *Agrobacterium tumefaciens* strain *EHA105* and transformed into the *bz1* mutant. For generation of rice *bz/cs1* mutants, specific single-guide RNA (sgRNA) targeting the *BZ1* gene was designed using the web-based tool CRISPR-P (http://www.genome.arizona.edu/crispr/) and assembled into the binary expression vector pRGEB32. For generation of *BZ1pro::GUS* transgenic plants, the putative promoter of BZ1 (1990 bp) was amplified and cloned into the pCAMBIA1301 vector upstream of the GUS reporter gene. To generate the BZ1-overexpressing plants, a 1227-bp cDNA fragment encoding full-length *BZ1* was amplified, and then cloned into the binary expression vector pCAMBIA1300s (driven by double 35S promoter). The recombinant constructs were introduced into *Agrobacterium tumefaciens* strain *EHA105* and transformed into calli of kitaake. The transgenic lines were grown in paddy fields in Shenyang Agricultural University, Shenyang, China, during the normal rice growing seasons. The phenotypes were detected in the homozygous T2 generation of transgenic plants.

### Subcellular Localization and GUS Activity Assays

To determine the exact subcellular localization of BZ1, the BZ1 cDNA was fused in-frame with EGFP and ligated into the pCAMBIA1302 vector. The expression construct was transfected into rice protoplasts, with EGFP alone as the control. After overnight incubation in the dark, the protoplasts expressing GFP were imaged by a confocal laser scanning microscope (LSM780, Zeiss, Germany) using 488 nm excitation and 500-530 nm emission pass-filters.

For GUS activity analysis, *BZ1pro:GUS* transgenic plants were used to detect GUS activity. Various rice organs and tissues were picked up and stained in 1 mM 5-bromo-4-chloro-3-indolyl-β-glucuronic acid in 50 mM sodium phosphate (pH 7.2), 0.1% Triton X-100, and 20% methanol for 12 h at 37 °C. After clearing in 95% ethanol, the samples were mounted in water and observed with a light microscope (Zeiss, Heidenheim, Germany).

### Generation of Recombinant BZ1 and Detection of its Enzymatic Activity

The coding sequence of *BZ1* was amplified using PCR and cloned into the pG21T vector (GST). The resulting plasmid was transformed into *E. coli* TOP10 and grown in LB medium, and IPTG (isopropyl-1-thio-b-D-galacto-pyranoside) was used to induce the expression of the recombinant protein. Recombinant BZ1 protein was affinity-purified through glutathione Sepharose resin (ProbeGene, Xuzhou, China). The molecular weight of GST-tagged BZ1 recombinant protein was predicted to be 74.8 kDa (https://web.expasy.org/compute_pi/). The enzyme activity assays were performed as previously described (Barber et al., 2006). Briefly, for UDP-Gal 4-epimerase assay, 150 μl of UDP-Gal (JK scientific, catalog number 137868-52-1, Beijing, China) solution was added to 50 μl of pre-warmed enzyme solution, containing 50 mM Tris/HCl (pH 7.6), 0.1 mM NAD, and 100 μg·ml^− 1^ acetylated BSA (Sigma catalog number B2518). After 15 min at 37 °C, the reaction was stopped by adding 25 μl of 1.5 M HCl. After 5 min at 100 °C and cooling, 25 μl of 1.5 M NaOH was added. For the UDP-Glc 4-epimerase assay, 150 μl of UDP-Glc (Sigma catalog number U4625) solution was added to 50 μl of pre-warmed enzyme solution, containing 50 mM Tris/HCl (pH 7.6), 0.1 mM NAD, and 100 μg·ml^− 1^ acetylated BSA (Sigma catalog number B2518). The epimerase reaction was stopped after 15 min at 37 °C with 25 μl of 1.5 M HCl, hydrolyzed at 100 °C for 5 min, and neutralized using a mole equivalent of NaOH. The content of generated glucose and galactose was quantified as previously described (Barber et al., 2006).

### Plant Cell Wall Fractionation and Determination

The plant cell wall fractionation procedure and total cellulose and hemicelluloses assay were conducted as previously reported (Li et al., 2015). Total lignin content including acid-insoluble (AIL) and acid-soluble lignin (ASL) were detected by the two-step acid hydrolysis method as described previously (Li et al., 2015). For monosaccharides composition analysis, the mature stem were ground into fine powder and washed in phosphate buffer (50 mM, pH 7) twice, extracted twice with 95% dimethyl sulfoxide at room temperature for 4 h, and then extracted twice with 75% ethanol at 60 °C for 2 h, and dried under vacuum. The dried destarched cell wall materials were hydrolyzed by incubation in 68% (v:v) H_2_SO_4_ at room temperature for 1 h and then in 2 M H_2_SO_4_ at 121 °C for 1 h. The hydrolyzed monosaccharides were analyzed by GCMS. All experiments were carried out in biological triplicate.

### Cellulose CrI and DP Detections

The lignocellulose crystallinity index (CrI) was detected by the X-ray diffraction method using Rigaku-D/ MAX instrument (Ultima III; Japan) as previously described (Zhang et al., 2013). The relative DP of cellulose was measured by the viscometry method as described previously (Zhang et al., 2013).

### Immunological and Chemical Analyses

The immunolocalization analyses were performed as previously described (Li et al., 2018) with specific monoclonal antibodies JIM13 and LM2. For histochemical staining, fresh second internodes (counting from the top) were cut and incubated in βGlcY [2 mg ml^− 1^ (β-D-glucosyl)_3_ in 0.1 M NaCl] (Biosupplies Australia, Cat#100-4) for 1 h (Yariv et al., 1962), and the reagent βManY [2 mg ml^− 1^ (β-D-mannosyl)_3_ in 0.1 M NaCl] (Biosupplies Australia, Cat#100-6) was the control. The sections were then visualized using a fluorescent microscope (Zeiss AX10).

For immunoelectron microscopy observation, nickel grids carrying ultrathin stem internode sections prepared from the booting stage were sequentially floated in 0.01 M sodium phosphate buffer (PBS, pH 7.2) containing 5% (w/v) bovine serum albumin (BSA) for 5 min, and then for 1 h at 37 °C in PBS containing diluted JIM13 or LM2. After several washes in PBS, ultrathin sections were incubated for 1 h at 37 °C in PBS containing goat anti-rat IgG antibody conjugated to 10-nm colloidal gold (1:40, Sigma-Aldrich, St. Louis, MO, USA). After several washes with PBS, ultrathin sections were washed with distilled water, air-dried, counterstained with 2% uranyl acetate, and examined via TEM (Hitachi H7700, Hitachi Ltd., Tokyo, Japan). Negative controls were established using the same procedure with pre-immune serum.

AGPs were isolated from the second stem internodes of wild-type and *bz1* plants at the booting stage as described previously (Kreuger and van Holst, 1993). The concentration of AGPs was determined by the single radial diffusion method developed by van Holst and Clarke (1985). Gum arabic (Biosupplies Australia, Cat#100-4) was used as a substrate to build standard curves. Monosaccharide composition of AGPs was determined by GCMS analysis (van Hengel and Roberts, 2002).

### Microscopic Observations

The second stem internode tissues (0.5 cm sections above the node) at the heading stage were used to observe cell wall structures by TEM (Hitachi H7700, Hitachi Ltd., Tokyo, Japan). For chloroplast structure observation, as shown in Fig. 1h and i, the corresponding parts of the leaves of wild-type and *bz1* plants at the tillering stage were harvested. The procedures of sample pretreatment and observation by TEM were performed as described in our previous report (Li et al., 2018)

For cryogenic scanning electron microscopy (Cryo-SEM) observation of cellulose orientation, the second stem internode tissues (0.5 cm sections above the node) at heading stage were harvested. The samples were rinsed in PBS and treated with Updegraff reagent (Updegraff, 1969; Zhang et al., 2010) at 100 °C for 60 min to remove non-crystallized polysaccharides and lignin. The samples were washed six times with 10 mL distilled water to remove Updegraff reagent. The cellulose microfibrils structure was examined using a Cryo-SEM (Regulus 8100, Hitachi Ltd., Tokyo, Japan). Five independent samples were scanned, and each was observed 5 to 10 times, and the representative image was used in this study.

### Measurement of Total Chlorophyll Content and Photosynthetic Characters

Total chlorophyll content was measured as described previously (Arnon, 1949), briefly, about 50 mg leaves were collected from top leaves at three developmental stages (tillering stage, jointing stage, heading stage) in 10 mL 96% ethanol, and total chlorophyll content was calculated spectrophotometrically based on the absorbance of the supernatant at 649 and 665 nm. Leaf photosynthetic characteristics were measured on top leaves of wild-type and *bz1* plants at three developmental stages with CIRAS-3 portable photosynthesis system (PP SYSTEMS CIRAS-3).

### Lipid Measurements

Freshly collected leaves from five individual plants at the tillering stage were weighed and inactivated with hot isopropanol, using a modified protocol as previously described (Welti et al., 2002). Following inactivation, the samples were incubated in extraction solvent containing chloroform:methanol:300 mM ammonium acetate (30:41.5:3.5) (v/v/v) at 25 °C for 24 h. After incubation, the samples were centrifuged and supernatants were transferred to fresh tubes. These steps were repeated once and lipid extracts from both rounds of extraction were pooled and dried on a SpeedVac (Genevac, UK). Lipid extracts were stored at − 80 °C until LCMS analyses. LCMS analyses were performed using an Exion UPLC coupled with Sciex QTRAP 6500 PLUS at LipidALL Technologies, as described previously (Gao et al., 2017).

### RNA Sequencing Analysis

Genome-wide expression studies using RNAseq analysis were performed with the wild-type and *bz1* independently grown under the same growth conditions. Total RNAs was extracted from the second internodes (counting from top) at the booting stage and top leaves at the tillering stage. RNA quality was determined using an Agilent 2100 Bioanalyzer (Agilent Technologies). Stranded RNA sequencing libraries were constructed from 2 μg of total RNAs using the KC-DigitalTM Stranded mRNA Library Prep Kit for Illumina® (Catalog NO. DR08502, Wuhan Seqhealth Co., Ltd. China) following the manufacturer’s instruction. The kit eliminates duplication bias in PCR and sequencing steps, by using a unique molecular identifier (UMI) of eight random bases to label the pre-amplified cDNA molecules. The library products were enriched, quantified, and deeply sequenced on a Hiseq X 10 sequencer (Illumina).

Raw sequencing data were first filtered by Trimmomatic (version 0.36), low-quality reads were discarded and the reads contaminated with adaptor sequences were trimmed. Clean Reads were further treated with in-house scripts to eliminate duplication bias introduced in library preparation and sequencing. Differentially expressed genes (DEGs) were identified by DEseq2 with the criteria of absolute log2 (fold change) ≥1 and FDR corrected *P*-value ≤0.05. The Kyoto Encyclopedia of Genes and Genomes (KEGG) enrichment analysis for differentially expressed genes was both implemented using KOBAS software (version: 2.1.1) with a corrected *P*-value cut-off of 0.05 to judge statistically significant enrichment.

### qRT-PCR Measurement

Total RNA extraction was conducted using the Total RNA Extraction Kit (TaKaRa) and first-strand cDNA was synthesized with the PrimeScript RT Master Mix (TaKaRa). Real-time RT-PCR was performed as described previously (Li et al., 2015) by using SYBR Premix Ex TaqII (TaKaRa) on an Applied Biosystems QuanStudio 3 Real-Time PCR System. The following standard thermal profile was used for all PCRs: 95 °C for 30 s, 40 cycles of 95 °C for 5 s, and 60 °C for 34 s. All reactions were done at least in triplicates. The primers used for qRT-PCR analysis were listed in Table S4.

## Supplementary information

**Additional file 1: Figure S1.** The *BZ1* gene is confirmed to be responsible for the brittle culm and zebra leaf phenotypes. (**a)** The transgenic plants *bz/cs1* generated by CRISPR/Cas9 approach had two base pairs deletion in the third exon of LOC_Os08g28730. The arrowhead indicates two base pairs deletion (showing in red letters) in the third exon inducing a premature translational stop codon (underlined). (**b–d)** The complementary lines showed restored phenotypes as wild-type (**b),** whereas the *bz/cs1* displayed brittle culm **(c)** and zebra leaf phenotypes **(d)** as similar as *bz1*. **Figure S2.** Phylogeny of UGE isoforms in rice and *Arabidopsis* based on maximum likelihood. **Figure S3.** Comparison of cell wall composition **(a)**, chlorophyll content **(b)** and photosynthetic efficiency **(c)** between WT and BZ1ox7 transgenic line. * indicates significant differences between WT and BZ1ox7 by *t*-test at *P* < 0.05. **Figure S4.** (**a)** Number of genes that are upregulated and downregulated in stem and leaf of *bz1* compared with those in the WT. (**b)** Relative expression level of *UGEs* and *PHD1* based on RNA-Sequencing analysis. (**c)** Relative expression level of *UGEs* and *PHD1* based on qRT-PCR assay. **Table S1.** Comparison of expression levels of putative AGPs biosynthesis-related genes between WT and *bz1* plants by stem RNA-Sequencing analysis. **Table S2.** Comparison of expression levels of leaf color associated genes between WT and *bz1* plants by leaf RNA-Sequencing analysis. **Table S3.** Significant alterations of genes involved in the photosynthesis pathway in comparison of *bz1* leaf RNA-Sequencing data to that of the WT. **Table S4.** Primers used for qRT-PCR analysis.

## Data Availability

The datasets supporting the conclusions of this article are included within the article and its additional files.
