# Supplementary material for: The Rice BZ1 Locus Is Required for Glycosylation of Arabinogalactan Proteins and Galactolipid and Plays a Role in both Mechanical Strength and Leaf Color
Source: Rice (N Y). 2020 Jun 17;13:41. doi: 10.1186/s12284-020-00400-9 (PMC7300173; doi:10.1186/s12284-020-00400-9)
Supplement: Supplementary file 1 — Additional file 1: Figure S1. The BZ1 gene is confirmed to be responsible for the brittle culm and zebra leaf phenotypes. (a) The transgenic plants bz/cs1 generated by CRISPR/Cas9 approach had two base pairs deletion in the third exon of LOC_Os08g28730. The arrowhead indicates two base pairs deletion (showing in red letters) in the third exon inducing a premature translational stop codon (underlined). (b–d) The complementary lines showed restored phenotypes as wild-type (b), whereas the bz/cs1 displayed brittle culm (c) and zebra leaf phenotypes (d) as similar as bz1. Figure S2. Phylogeny of UGE isoforms in rice and Arabidopsis based on maximum likelihood. Figure S3. Comparison of cell wall composition (a), chlorophyll content (b) and photosynthetic efficiency (c) between WT and BZ1ox7 transgenic line. * indicates significant differences between WT and BZ1ox7 by t-test at P < 0.05. Figure S4. (a) Number of genes that are upregulated and downregulated in stem and leaf of bz1 compared with those in the WT. (b) Relative expression level of UGEs and PHD1 based on RNA-Sequencing analysis. (c) Relative expression level of UGEs and PHD1 based on qRT-PCR assay. Table S1. Comparison of expression levels of putative AGPs biosynthesis-related genes between WT and bz1 plants by stem RNA-Sequencing analysis. Table S2. Comparison of expression levels of leaf color associated genes between WT and bz1 plants by leaf RNA-Sequencing analysis. Table S3. Significant alterations of genes involved in the photosynthesis pathway in comparison of bz1 leaf RNA-Sequencing data to that of the WT. Table S4. Primers used for qRT-PCR analysis. [file 12284_2020_400_MOESM1_ESM.pdf]

Supplemental figures and tables

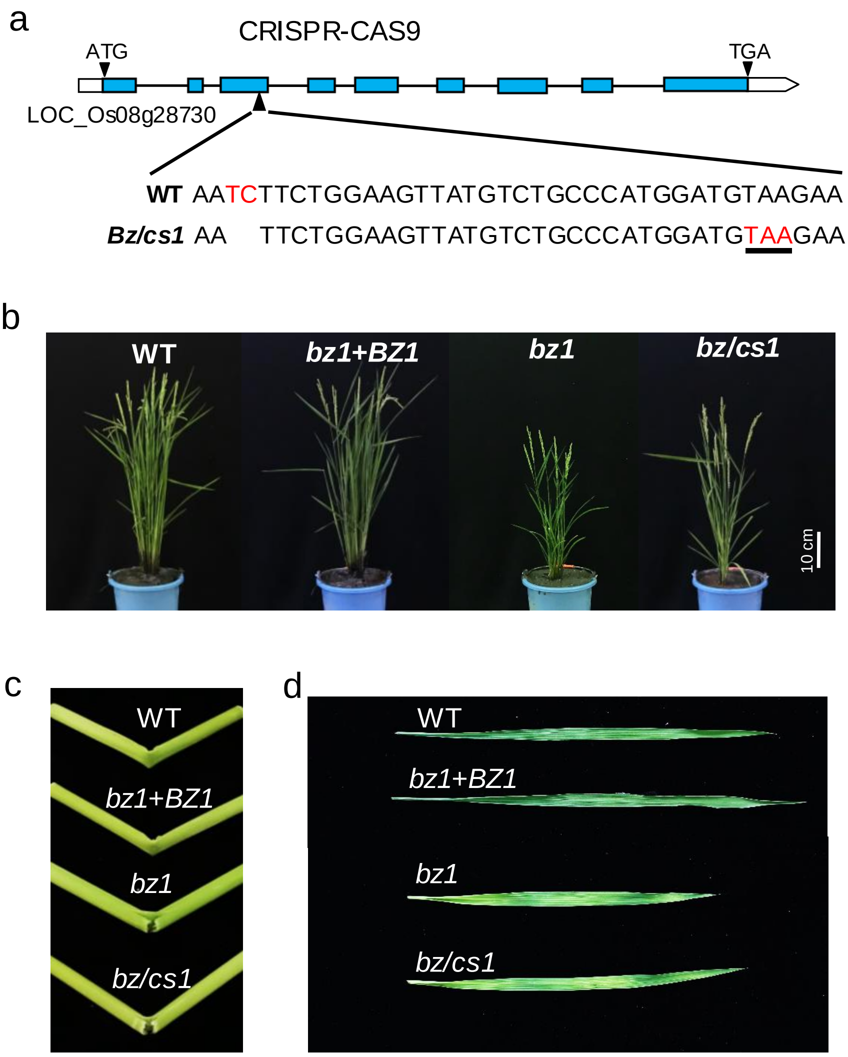

Figure S1. The *BZ1* gene is confirmed to be responsible for the brittle culm and zebra leaf phenotypes. **a,** The transgenic plants *bz/cs1* generated by CRISPR/Cas9 approach had two base pairs deletion in the third exon of LOC_Os08g28730. The arrowhead indicates two base pairs deletion (showing in red letters) in the third exon inducing a premature translational stop codon (underlined). **b–d,** The complementary lines showed restored phenotypes as wild-type (**b**) whereas the *bz/cs1* displayed brittle culm (**c**) and zebra leaf phenotypes (**d**) as similar as *bz1*.

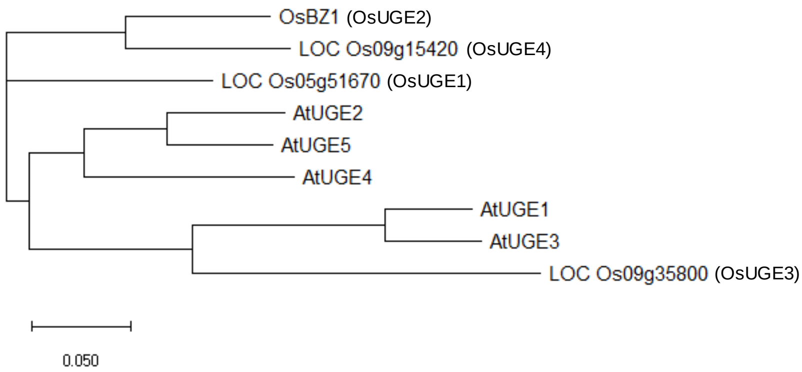

Figure S2. Phylogeny of UGE isoforms in rice and *Arabidopsis* based on maximum likelihood.

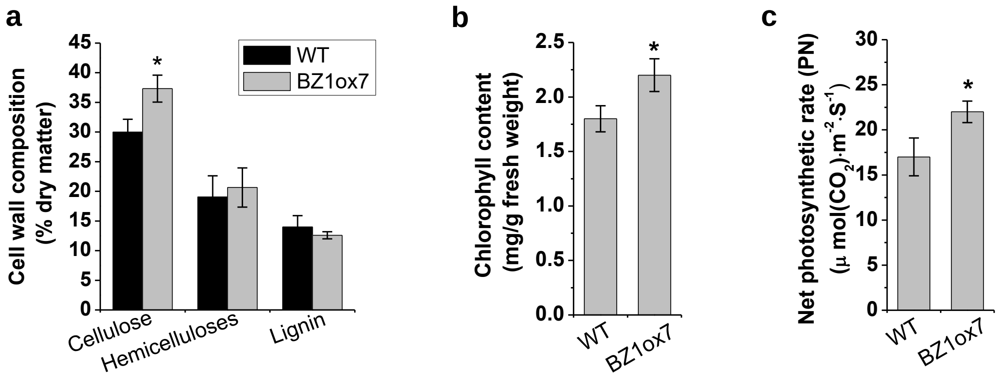

Figure S3. Comparison of cell wall composition **(a)**, chlorophyll content **(b)** and photosynthetic efficiency **(c)** between WT and BZ1ox7 transgenic line * indicates significant differences between WT and BZ1ox7 by *t*-test at $P < 0.05$.

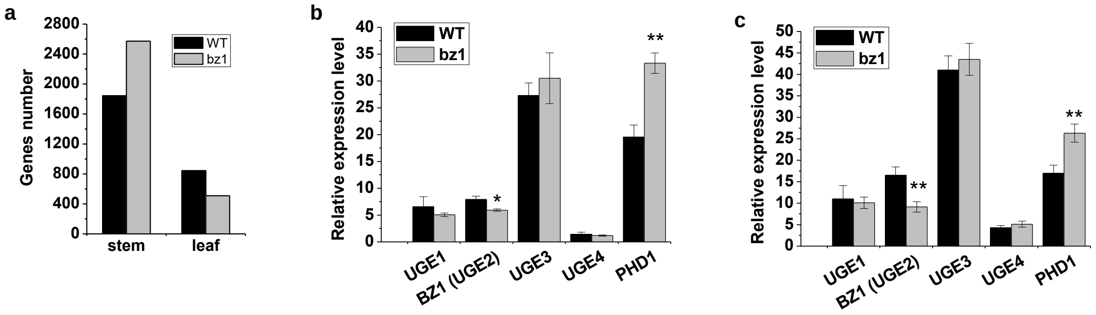

Figure S4. **a,** Number of genes that are upregulated and downregulated in stem and leaf of *bz1* compared with those in the WT. **b,** Relative expression level of *UGEs* and *PHD1* based on RNA-Sequencing analysis. **c,** Relative expression level of *UGEs* and *PHD1* based on qRT-PCR assay.

Table S1. Comparison of expression levels of putative AGPs biosynthesis-related genes between WT and *bz1* plants by stem RNA-Sequencing analysis.

Name	Locus	RPKM		$\log_2$ Ratio	*p*	FDR	change
		WT	*bz1*				
AGP1	LOC_Os08g37630	11.51	3.28	-1.62	0.00	0.01	down
AGP2	LOC_Os01g71170	98.80	48.70	-0.85	0.00	0.01	down
AGP3	LOC_Os03g08940	103.99	77.57	-0.19	0.76	0.86	
AGP12	LOC_Os01g02010	132.41	194.44	0.74	0.01	0.03	up
AGP14	LOC_Os01g37950	448.79	261.87	-0.59	0.03	0.09	down
AGP17	LOC_Os01g55220	35.09	3.39	-3.18	0.00	0.00	down
AGP19	LOC_Os01g57040	24.39	29.73	0.50	0.12	0.25	
AGP20	LOC_Os02g16500	278.33	115.58	-1.07	0.00	0.00	down
AGP21	LOC_Os02g48710	614.19	1155.84	1.10	0.00	0.00	up
AGP23	LOC_Os05g12580	285.37	162.80	-0.60	0.09	0.20	
AGP24	LOC_Os06g21400	48.10	197.49	2.30	0.03	0.10	up
AGP26	LOC_Os06g30920	22.95	21.04	0.09	0.78	0.87	
AGP27	LOC_Os07g38630	44.06	47.76	0.35	0.42	0.59	
AGP29	LOC_Os01g42210	1.77	0.83	-0.90	0.04	0.12	down
AGP31	LOC_Os03g63540	26.13	30.61	0.42	0.14	0.28	
LLA1	LOC_Os03g26820	26.43	17.41	-0.42	0.14	0.28	
LLA6	LOC_Os07g07790	0.59	2.22	2.07	0.01	0.05	up
LLA7	LOC_Os07g43290	15.56	10.96	-0.33	0.27	0.44	
FLA1	LOC_Os04g48490	10.48	1.17	-3.00	0.00	0.00	down
FLA2	LOC_Os03g03600	33.55	47.08	0.67	0.01	0.05	up
FLA3	LOC_Os08g23180	93.72	116.95	0.51	0.06	0.15	
FLA4	LOC_Os08g38270	63.22	77.08	0.50	0.11	0.24	
FLA5	LOC_Os08g39270	67.26	28.85	-1.04	0.00	0.00	down
FLA6	LOC_Os05g48900	254.09	327.34	0.56	0.04	0.11	up
FLA7	LOC_Os01g47780	354.01	372.81	0.27	0.31	0.49	
FLA8	LOC_Os01g06580	150.68	208.37	0.65	0.02	0.06	up
FLA9	LOC_Os05g07060	180.56	259.12	0.69	0.01	0.04	up
FLA10	LOC_Os09g30010	3.41	1.33	-1.17	0.00	0.02	down
FLA11	LOC_Os09g07350	158.94	296.77	1.09	0.00	0.00	up
FLA12	LOC_Os01g62380	7.90	4.93	-0.50	0.10	0.22	
FLA15	LOC_Os02g20560	4.13	12.81	1.82	0.00	0.00	down
FLA16	LOC_Os07g06680	301.56	464.02	0.81	0.00	0.02	up
FLA17	LOC_Os03g57490	0.77	0.68	0.04	0.93	0.96	
FLA21	LOC_Os02g49420	2.23	0.19	-3.37	0.00	0.00	down
FLA24	LOC_Os03g57460	100.11	175.04	1.02	0.01	0.03	
FLA26	LOC_Os05g38500	4.90	5.16	0.26	0.41	0.59	
FLA27	LOC_Os09g30486	78.36	96.39	0.49	0.07	0.17	
ELA2	LOC_Os02g06670	4.73	3.36	-0.32	0.36	0.53	

Table S2. Comparison of expression levels of leaf color associated genes between WT and *bz1* plants by leaf RNA-Sequencing analysis.

Gene	Locus	WT	*bz1*	logFC	*P* Value	FDR	Change
D1; RGA1; D89	LOC_Os05g26890	62.91	75.88	0.27	0.11	0.35	
Spl11	LOC_Os12g38210	19.83	18.05	-0.14	0.43	0.71	
spl5; SF3b3; 0sSL5	LOC_Os07g10390	7.11	6.70	-0.09	0.62	0.84	
Se5; OsHY1; OsHO1; ygl2; grc1	LOC_Os06g40080	167.01	159.34	-0.06	0.71	0.88	
OsDVR	LOC_Os03g22780	68.84	83.87	0.29	0.08	0.28	
YGL1; ygl80	LOC_Os05g28200	80.51	118.72	0.56	0.00	0.03	Up
OsChlH	LOC_Os03g20700	323.51	594.44	0.88	0.00	0.00	Up
OsCAO1; PGL	LOC_Os10g41780	44.14	80.68	0.87	0.00	0.01	Up
NAL9; VYL; ClpP	LOC_Os03g29810	41.60	55.31	0.41	0.02	0.12	
Chl1; OsChlD; ygl3; Ygl7; ygl98	LOC_Os03g59640	156.92	236.01	0.59	0.00	0.01	Up
Chl9; OsChlI	LOC_Os03g36540	210.19	316.47	0.59	0.00	0.01	Up
NYC1	LOC_Os01g12710	434.41	300.22	-0.53	0.00	0.01	Down
SGR	LOC_Os09g36200	1.40	2.31	0.72	0.03	0.14	
v2	LOC_Os03g20460	12.16	13.91	0.19	0.35	0.64	
OsCRTISO; ZEBRA2; PHS3; ZL2; MHZ5	LOC_Os11g36440	40.23	42.59	0.08	0.63	0.84	
NOL	LOC_Os03g45194	13.52	33.08	1.28	0.00	0.00	Up
OsDOS	LOC_Os01g09620	12.17	7.01	-0.80	0.00	0.00	Down
OsGluRS; Cde1(t)	LOC_Os02g02860	28.12	48.28	0.77	0.00	0.01	Up
OsHAP3A	LOC_Os01g61810	9.75	9.20	-0.08	0.69	0.87	
OsHAP3B	LOC_Os05g38820	9.25	9.93	0.10	0.61	0.83	
NYC3	LOC_Os06g24730	6.80	7.55	0.15	0.39	0.68	
OsMADS26	LOC_Os08g02070	3.22	17.99	2.47	0.00	0.00	Up
RNRL1; V3	LOC_Os06g07210	2.59	2.65	0.03	0.91	0.97	
RNRS1; St1	LOC_Os06g14620	5.44	5.95	0.13	0.73	0.89	
OsPHR2	LOC_Os07g25710	17.64	17.19	-0.04	0.82	0.94	
OsFRDL1	LOC_Os03g11734	0.83	0.68	-0.28	0.37	0.66	
OsSPX1	LOC_Os06g40120	32.54	24.29	-0.42	0.25	0.54	
SPP	LOC_Os06g41990	226.97	224.20	-0.02	0.91	0.97	
DTH8; Ghd8; OsHAP3H; LHD1; EF8	LOC_Os08g07740	8.58	6.71	-0.35	0.10	0.32	
OsLSD1; OsLOL1	LOC_Os08g06280	38.03	33.36	-0.19	0.25	0.54	
SPL3; OsEDR1; OsACDR1; OsMAPKKK1	LOC_Os03g06410	14.58	16.82	0.20	0.23	0.51	
β-OsLCY; zebra524	LOC_Os02g09750	73.82	66.24	-0.15	0.41	0.69	
RLIN1; LLM1	LOC_Os04g52130	165.23	250.86	0.60	0.00	0.03	
etl1	LOC_Os11g01210	69.25	43.88	-0.65	0.00	0.03	
etl2	LOC_Os12g01210	186.70	129.89	-0.52	0.02	0.10	
OsAPX2; APXb	LOC_Os07g49400	208.22	117.78	-0.82	0.01	0.08	
OsWRKY53	LOC_Os05g27730	47.34	72.62	0.61	0.00	0.05	Up
WSL12; OsNDPK2	LOC_Os12g36194	23.57	45.04	0.93	0.00	0.03	Up
RLS1	LOC_Os02g10900	2.61	6.02	1.20	0.00	0.00	Up
OsABC1-2	LOC_Os02g36570	121.91	144.37	0.24	0.12	0.37	
CHR729; CRL6	LOC_Os07g31450	14.61	14.74	0.01	0.95	0.98	
OsNUS1;V1	LOC_Os03g45400	106.32	109.78	0.04	0.78	0.92	
YSA	LOC_Os03g40020	18.08	15.27	-0.25	0.13	0.38	
OsHPL3; cea62	LOC_Os02g02000	39.07	51.38	0.39	0.09	0.31	
MPR25	LOC_Os04g51350	13.63	18.07	0.40	0.02	0.11	
YLC1; OsV5A	LOC_Os09g21250	18.17	32.06	0.81	0.00	0.02	Up
OsPORB; FGL; PGL10	LOC_Os10g35370	184.67	258.38	0.48	0.01	0.07	
Cga1	LOC_Os02g12790	9.81	13.39	0.44	0.05	0.21	
PAPST1	LOC_Os01g16040	22.93	31.30	0.44	0.01	0.07	
WSL	LOC_Os01g37870	21.42	26.35	0.29	0.08	0.28	
OsHO2; OsYLC2	LOC_Os03g27770	34.88	61.14	0.80	0.00	0.01	Up
HYR	LOC_Os03g02650	0.78	0.93	0.22	0.58	0.81	
AM1	LOC_Os04g58620	52.91	56.46	0.10	0.55	0.79	
OsPPR1	LOC_Os09g24680	1.42	1.03	-0.46	0.11	0.35	
OsPDF1B	LOC_Os01g45070	59.02	57.06	-0.05	0.76	0.90	
SPL29; UAP1	LOC_Os08g10600	6.25	6.93	0.15	0.44	0.72	
SPL33	LOC_Os01g02720	11.81	11.37	-0.05	0.76	0.91	
WLP1	LOC_Os01g54540	339.98	568.88	0.74	0.00	0.01	Up
OsRH34	LOC_Os03g36930	9.04	13.56	0.58	0.00	0.03	Up
OsV5B	LOC_Os08g32130	2.91	2.66	-0.13	0.63	0.84	
OsCUL3a	LOC_Os02g51180	5.51	5.39	-0.03	0.88	0.96	
LYL1	LOC_Os02g51080	740.99	830.74	0.17	0.35	0.64	
AL2; CRS1	LOC_Os09g19850	41.61	51.25	0.30	0.06	0.25	
ASL2; RPL21c	LOC_Os02g15900	330.28	616.38	0.89	0.00	0.01	Up
LLB	LOC_Os07g14350	18.66	27.94	0.58	0.00	0.01	Up
YGL138(t)	LOC_Os11g05552	56.33	80.27	0.51	0.00	0.03	Up
OsCYO1	LOC_Os09g28480	13.43	11.46	-0.23	0.18	0.45	
RLS3	LOC_Os03g38990	24.46	27.32	0.16	0.35	0.64	
OsNaPRT1; LT S1	LOC_Os03g62110	22.93	16.73	-0.45	0.01	0.07	
BGL11(t)	LOC_Os11g38040	2.80	2.43	-0.20	0.49	0.76	
TCD5	LOC_Os05g34040	17.90	25.16	0.49	0.01	0.08	
YSS1	LOC_Os04g59570	214.49	241.29	0.17	0.29	0.58	
GIC	LOC_Os04g57920	3.57	2.23	-0.67	0.01	0.06	
Fd-GOGAT1; lc7; ABC1; spl23	LOC_Os07g46460	628.62	669.76	0.09	0.56	0.80	
LMR; LRD6-6	LOC_Os06g03940	12.14	13.58	0.16	0.37	0.66	
OsGATA12	LOC_Os03g61570	9.32	8.42	-0.14	0.46	0.74	
OsFdC2	LOC_Os03g48040	205.38	216.38	0.07	0.64	0.85	
OsPAP1/OspTAC3; WSL3	LOC_Os10g32540	48.94	79.35	0.69	0.00	0.02	Up
OsAld-Y; ygdl-1	LOC_Os06g40640	289.66	187.83	-0.62	0.00	0.04	Down
TCD10	LOC_Os10g28600	81.80	98.94	0.27	0.09	0.30	
WSP1	LOC_Os04g51280	331.01	354.71	0.10	0.53	0.78	
YGL8	LOC_Os01g17170	356.29	600.84	0.75	0.00	0.01	Up
YL1	LOC_Os02g05890	53.18	96.68	0.86	0.00	0.00	
OsDG2	LOC_Os02g39340	20.21	28.89	0.51	0.01	0.05	
OsValRS2; WP1	LOC_Os07g06940	31.45	50.24	0.67	0.00	0.02	Up
AL1	LOC_Os03g31150	61.89	81.98	0.40	0.01	0.08	
GRY79	LOC_Os02g33610	191.54	189.43	-0.02	0.91	0.97	
OsABCI8	LOC_Os11g29850	7.89	8.68	0.14	0.48	0.75	

Table S3. Significant alterations of genes involved in photosynthesis pathway in comparison of *bz1* leaf RNA-Sequencing data to that of the WT.

Accession	WT	*bz1*	Log2 Ratio	*P*	FDR	Description
LOC_Os01g57962	3.58	1.69	-1.08	0.04	0.18	photosystem I P700 chlorophyll a apoprotein A2, putative, expressed
LOC_Os01g57964	2.36	1.01	-1.22	0.02	0.12	photosystem I P700 chlorophyll a apoprotein A1, putative, expressed
LOC_Os03g55874	4.99	1.99	-1.32	0.03	0.17	ATP synthase subunit beta, putative, expressed
LOC_Os04g16748	166.12	69.50	-1.26	0.00	0.03	ATP synthase B chain, putative, expressed
LOC_Os04g16740	71.11	31.80	-1.17	0.00	0.04	ATP synthase subunit alpha, putative, expressed
LOC_Os04g16872	8.81	3.69	-1.25	0.02	0.12	photosystem II D2 protein, putative, expressed
LOC_Os05g01675	8.30	3.41	-1.29	0.03	0.15	photosystem I P700 chlorophyll a apoprotein A1, putative, expressed
LOC_Os06g02980	1.35	0.44	-1.60	0.00	0.03	ATP synthase F1, epsilon subunit family protein, expressed
LOC_Os06g39756	3.74	1.06	-1.78	0.00	0.04	ATP synthase epsilon chain, putative, expressed
LOC_Os07g05360	1.16	0.34	-1.77	0.00	0.03	photosystem II 10 kDa polypeptide, chloroplast precursor, putative, expressed
LOC_Os07g22498	32.34	15.45	-1.07	0.00	0.04	photosystem I iron-sulfur center, putative, expressed
LOC_Os10g21230	11.36	4.83	-1.22	0.04	0.18	ATP synthase C chain, putative, expressed
LOC_Os10g21238	6.49	2.49	-1.38	0.02	0.13	ATP synthase B chain, putative, expressed
LOC_Os10g21240	16.66	7.25	-1.20	0.00	0.02	ATP synthase, putative, expressed
LOC_Os10g21250	1.81	0.73	-1.29	0.02	0.11	photosystem I P700 chlorophyll a apoprotein A1, putative, expressed
LOC_Os10g21266	2.89	1.04	-1.46	0.01	0.08	ATP synthase subunit beta, putative, expressed
LOC_Os10g21406	13.92	5.60	-1.32	0.00	0.03	photosystem I iron-sulfur center, putative, expressed
LOC_Os10g38229	3.08	1.14	-1.44	0.01	0.06	photosystem I P700 chlorophyll a apoprotein A1, putative, expressed
LOC_Os10g38272	88.32	32.73	-1.44	0.00	0.01	ATP synthase B chain, putative, expressed
LOC_Os10g39880	9.09	2.70	-1.73	0.05	0.20	photosynthetic reaction center protein, putative, expressed
LOC_Os10g41689	1.72	0.53	-1.69	0.01	0.07	photosystem II D2 protein, putative, expressed

Table S4. Primers used for qRT-PCR analysis

Gene	Locus	Annotation	Forward primers	Reverse primers
Actin1	LOC_Os03g50885	Actin	ACCATTGGTGCTGAGCGTTT	CGCAGCTTCCATTCCTATGAA
UGE1	LOC_Os05g51670	UDP-Glu/Gal epimerase 1	TGGAACCGACTACAACACCA	CAGCAAATACAAGCGGGATT
BZ1 (UGE2)	LOC_Os08g28730	UDP-Glu/Gal epimerase 2	CTAAAAGCTGTGGGCGAAAG	TGCCATATGGATTGTTTGGA
UGE3	LOC_Os09g35800	UDP-Glu/Gal epimerase 3	CCCAACAACCTTCTTCCGTA	AAGCCACACAACCAATGTCA
UGE4	LOC_Os09g15420	UDP-Glu/Gal epimerase 4	CGTTCATTTTGCTGGTCTGA	ACGGCAAATCTCCTCTGCTA
AGP1	LOC_Os08g37630	Arabinogalactan protein 1	TGGCCATGGCCGCGCTCTT	AAACCGGGGGAGGCGTCGT
AGP2	LOC_Os01g71170	Arabinogalactan protein 2	TTCCTCCTCGTACTCGTGCT	GGAGCAGCGGCATTATGT
AGP14	LOC_Os01g37950	Arabinogalactan protein 14	ATGGCCAGGTTCTCCGCA	AAATACACAGATCCCAAACC
AGP17	LOC_Os01g55220	Arabinogalactan protein 17	AAGGCCTTGGCTCTCCTCT	GAAGGCCAGGAGCGAGAC
AGP20	LOC_Os02g16500	Arabinogalactan protein 20	CTCGTCGTCGGCATCTTC	GGGAGAAGAAGCATCCAGTG
AGP29	LOC_Os01g42210	Arabinogalactan protein 29	GCCTTGTTTGGCATCAATTT	TGGCAGTGACTTGAGGTCTG
FLA1	LOC_Os04g48490	Fasciclin-like AGP1	AAGAAGCTGCACTCCCTCAG	CCTCAAGAACGGAGAGGTTG
FLA5	LOC_Os08g39270	Fasciclin-like AGP5	GAATCCAAAGCAGCCATGAT	GTCCGTCTTCTCCAGGAGGT
FLA10	LOC_Os09g30010	Fasciclin-like AGP10	GTTCCTGCCCAGGTACAAGA	GTTCTGCACCGTGAAGTTGT
FLA21	LOC_Os02g49420	Fasciclin-like AGP21	CCCTACAACATCTCCGTGCT	TTCTTCTTGCCACCATCTCC
DVR	LOC_Os03g22780	Divinyl-reductase	CCGTCCTTCACCTACAGCAT	CGATGGGGAGGATCTTGTT
YGL1	LOC_Os05g28200	Prenyltransferase	TTGGGCACTGTTGTTAGCAG	CAATGTAGCTCGCACCAAGA
CAO1	LOC_Os10g41780	Chlorophyllide a oxygenase	GCTGCTCTACCGGATGTCTC	ACAACCGATACCGGATACCA
ClpP	LOC_Os03g29810	Clp protease	GATCAAGCCCAAAGTTGGAA	CCAAGGTTTGCCCAGTAAAA
ChlD	LOC_Os03g59640	Magnesium-chelatase subunit chlD	AAAGAATGGCTCGAAAAGCA	GCCACCACATGGTAGCTTCT
ChlH	LOC_Os03g20700	Magnesium-chelatase subunit chlH	TGGAGCAGCTCTGCACTAGA	TTGGAGGACGGAGTAGATGG
ChlI	LOC_Os03g36540	Magnesium-chelatase subunit chlI	ATGGAACACCGTGGAGAGAG	GCTGGAGCTTGTCTTGTTCC
SGR	LOC_Os09g36200	Senescence-inducible chloroplast stay-green protein 1	AGGGGTGGTACAACAAGCTG	GCTCCTTGCGGAAGATGTAG
V1	LOC_Os03g45400	Antitermination NusB domain	TTTAGTGGCTGTTGCCAGTG	TCCATTGATTACCCGAGGAG
V2	LOC_Os03g20460	Guanylate kinase	AAGGAGCAGCAACCTTGAGA	GCGTTTCACCTCTTCTCTGG
NOL	LOC_Os03g45194	Oxidoreductase	TGGTGCAACGAAGAGAAGTG	ACGAGATAATCCGCAACCAC
GluRS	LOC_Os02g02860	Glutamyl-tRNA synthetase	ATTTGGCACTTCTTGGTTGG	AATGCCTGTGTCCTTCCATC
